# Novel mRNA biomarker-based liquid biopsy for the detection of resectable pancreatic cancer

**DOI:** 10.1186/s12885-025-14124-w

**Published:** 2025-04-23

**Authors:** Jong-chan Lee, Sung Won Kang, Eun-Jin Sim, Jin-Sik Bae, Seong-mo Koo, Mun-sub Byoun, Serin Kwon, Seoi Hong, Yunji Kim, Yuna Youn, Kwangrok Jung, Jaihwan Kim, Hyoung Hwa Jeong, Jihie Kim, Jin-Hyeok Hwang

**Affiliations:** 1 Department of Internal Medicine, Seoul National University Bundang Hospital, Seongnam, Korea; 2 Department of Internal Medicine, Seoul National University College of Medicine, Seoul, Korea; 3 Research Center, HuVet bio Inc, Seoul, Korea; 4 Department of Translational Medicine, Seoul National University College of Medicine, Seoul, Korea; 5 Department of Life Science, Dongguk University, Seoul, Korea; 6 Department of Computer and Artificial Intelligence, Dongguk University, Seoul, Korea

**Keywords:** Liquid biopsy, mRNA, Buffy coat, Pancreatic cancer, Biomarker

## Abstract

**Background:**

Pancreatic ductal adenocarcinoma (PDAC) is one of the most lethal malignancies and most often diagnosed at an advanced stage. Identification of markers for the early diagnosis of PDAC is crucial. In this study, we aimed to identify novel mRNA biomarkers for diagnosing PDAC, focusing on early-stage tumorigenesis and associated immunological changes.

**Methods:**

Blood samples and clinical information from 1,963 individuals were obtained from a single tertiary hospital between 2015 and 2021. Candidate mRNA biomarkers were identified through literature review, and their expression levels in buffy coat samples were measured using reverse-transcription quantitative polymerase chain reaction. Machine learning-based feature selection confirmed the final biomarker panel, which was tested using an independent dataset for diagnostic performance.

**Results:**

In total, 1,504 individuals (417 patients with PDAC and 1,087 non-disease controls) were eligible for the study. Among the 55 candidate biomarkers identified, 15 mRNAs (*CCL5*,* CCR5*,* CLEC7A*,* CXCL8*,* CXCR2*,* CXCR4*,* FOXP3*,* IFNA1*,* IFNL1*,* PTGES*,* PTGES2*,* PTGS2*,* SLC27A2*,* TNF*, and *VEGFA)* were selected based on their diagnostic performance in distinguishing PDAC from control groups. The final model, HELP-15 (Human Early Liquid biopsy for PDAC), identified all PDAC stages (area under the curve [AUC] = 0.956) in the test set. For resectable pancreatic cancer (RPC), the AUC was 0.968, compared to 0.910 for carbohydrate antigen 19 − 9 (CA19-9). The combined model of the panel and CA19-9 achieved an AUC of 0.985 in patients with RPC. For all PDAC stages in patients with normal CA19-9 levels, the AUC of the panel was 0.967, whereas CA19-9 alone or in combination with the panel had AUCs of 0.658 and 0.885, respectively.

**Conclusion:**

Compared to CA19-9, the mRNA biomarker panel, HELP-15, improved diagnostic performance in patients with RPC, particularly in those with normal CA19-9 levels.

**Supplementary Information:**

The online version contains supplementary material available at 10.1186/s12885-025-14124-w.

## Introduction

Pancreatic ductal adenocarcinoma (PDAC) is one of the most lethal malignancies among all cancer types, with the lowest 5-year survival rate, and its incidence is projected to increase continuously [1]. The high mortality observed in PDAC results from the difficulty of early diagnosis, the aggressive tumor biology, treatment-resistant tumor microenvironment (TME), and early metastatic propensity [2]. Because the prognosis of PDAC varies according to the initial staging and more than 85% of patients are diagnosed at an advanced stage, “early detection of resectable PDAC” is a major strategy in curing PDAC patients [3, 4]. In the same context, since recurrence of PDAC after surgical resection is still high, the “early detection of recurrence” is also an important issue in PDAC treatment [5]. This highlights the importance of developing effective biomarkers for the detection of early-stage PDAC.

Currently, the only FDA-approved clinical biomarker of PDAC is serum carbohydrate antigen 19 − 9 (CA19-9) [6]. Although CA19-9 is useful for determining treatment response, it has been known to be less suitable for early-stage diagnosis [7, 8]. Additionally, CA19-9 expression is genetically absent in Lewis antigen-negative individuals, comprising approximately 10% of the population [9]. Consequently, various biomarkers have been evaluated as alternatives to CA19-9 [10–13]. However, these alternative markers have neither demonstrated diagnostic performance superior to that of CA19-9 nor have they established their clinical reproducibility.

PDAC is immunosuppressive, similar to other cancer types, in which tumor cells modify the surrounding tumor microenvironment (TME) to evade host immune surveillance. Although the exact mechanism has not yet been defined, studies have shown that this process is highly correlated with the formation of tumor-associated mesenchymal stem cells, granulocyte macrophage colony-stimulating factor (GM-CSF), and polymorphonuclear myeloid-derived suppressor cells, which lead to alterations in immune cell responses and relevant signaling pathways [14–18]. We hypothesized that immune reprogramming by tumor cells influences immune system-related protein expression at the mRNA level during early tumorigenesis. Thus, detecting these biomolecular changes through liquid biopsy may facilitate early-stage PDAC diagnosis.

In this study, we aimed to develop a novel mRNA-based liquid biomarker panel as an alternative marker or additive marker of conventional CA19-9 in detecting early staged of PDAC, emphasizing the early tumorigenesis associated with immune reprogramming.

## Materials and methods

Samples were obtained from the Human Bioresource Center of the Seoul National University Bundang Hospital (SNUBH) between September 2015 and December 2021. The study was reviewed and approved by the Institutional Review Board (IRB approval number: X-2011-651-903) and was conducted in accordance with the Declaration of Helsinki. Private patient information was anonymized and de-identified prior to analysis.

### Patient recruitment

The clinical information of the patients and buffy coat samples were obtained from the electronic medical data and SNUBH biobank using the following inclusion criteria [1]. PDAC group: individuals who were histologically diagnosed with PDAC, including resectable pancreatic cancer (RPC), borderline resectable pancreatic cancer (BRPC), locally advanced pancreatic cancer (LAPC), and metastatic pancreatic cancer (MPC). Tumor stage was determined according to clinical staging system, since clinical staging is more commonly used in evaluating pancreatic cancer rather than TNM staging [19].

In this study, patients with PDAC were divided into two groups: RPC and advanced pancreatic cancer (APC), which included BRPC, LAPC, and MPC. The BRPC patients were classified into APC group with two following reasons. First, since 2016, the NCCN guidelines excluded upfront surgery as the first choice in treating BRPC whereas chemotherapy has been recommended, which means initial treatment option for BRPC is along with that for LAPC and MPC [2]. Second, since the focus of this study is on biomarkers for “early detection,” we adopted a stricter definition of RPC, excluding BRPC.

Non-disease control group was defined as follows: (1) individuals with no prior history of malignancy, AND (2) individuals who have well-known risk factors (e.g., chronic pancreatitis, type 2 diabetes) but do not have pancreatic cancer at the time of evaluation, OR (3) healthy controls confirmed to have no other diseases based on routine health check-ups.

Additionally, all samples were divided into CA19-9 low (< 37.0 U/mL) and high (≥ 37.0 U/mL) groups, according to the patients’ medical records, to further analyze the efficacy of candidate markers against individuals with normal levels of CA19-9.

Some patient data were excluded during medical record validation and reverse-transcription quantitative polymerase chain reaction (RT-qPCR) analysis. The exclusion criteria were as follows: (1) samples without complete medical records (e.g., CA19-9 value); (2) more than one month gap between the initial diagnosis date and the sample collection date; (3) samples collected after surgery or chemotherapy; and (4) samples of insufficient quality for RT-qPCR analysis (e.g., insufficient concentration, volume, or purity).

### Blood collection and RNA Preparation

Prior to buffy coat extraction, whole blood was collected in EDTA tubes. Whole blood samples were then centrifuged at 1,800 × *g* for 10 min at 4 °C within 4 h of collection. Buffy coat samples were separated from the plasma and red blood cell layers and stored at − 80 °C immediately after separation. Total RNA was isolated from buffy coat samples using a NucleoSpin RNA Blood kit (MACHEREY-NAGEL, Düren, Germany). For cDNA synthesis, 1 µg of total RNA was reverse-transcribed using the GoScript Reverse Transcription System (Promega, Madison, WI, USA). The cDNA product obtained was stored at − 80 °C.

### RT-qPCR assay

RT-qPCR was performed using a probe-based multiplex assay. Each probe was labeled at the 5′-end with a reporter dye (FAM or HEX). The primers and probes used in this study were obtained from Integrated DNA Technologies Inc. (Coralville, IA, USA). The mRNA expression levels were measured using GoTaq Probe qPCR Master Mix (Promega) in a 20 µL final volume. qPCR was performed using the QuantStudio 3 and QuantStudio 5 Real-Time PCR systems (Applied Biosystems, Foster City, CA, USA) under standard cycling conditions. The relative gene expression values were calculated by subtracting the *GAPDH* Ct value from the target gene Ct values, which were denoted as ΔCt:

$$\Delta Ct\, = \,C{t_{target{\text{ }}gene}}\, - C{t_{GAPDH}}$$

### Candidate marker selection

**Fig. 1:**
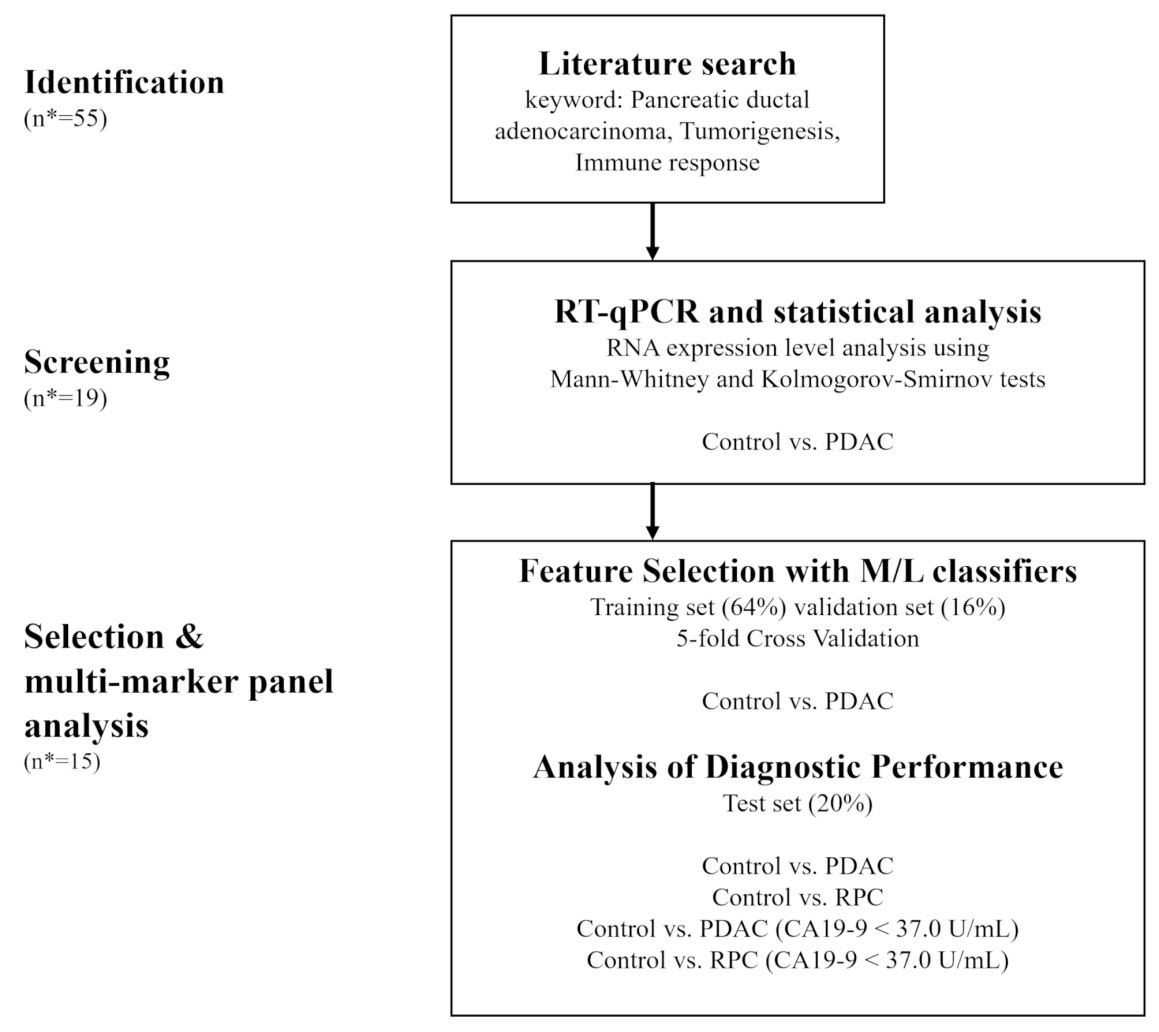
Schematic representation of marker selection and model construction. A literature search emulating PRISMA flowchart was conducted and 55 candidate markers were identified. Nineteen markers were identified using RT-qPCR and statistical analyses. Machine learning-based feature selection was conducted to generate optimal diagnostic models. Multi-marker models were evaluated in four comparisons based on sensitivity, specificity, and AUC. *n, the number of markers remaining after each process. AUC, area under the curve; RT-qPCR, reverse-transcription quantitative polymerase chain reaction

Although this is not a conventional systematic review and meta-analysis, we attempted to emulate the methodology outlined in the PRISMA flowchart, and searched PubMed and MEDLINE databases using the following query: (pancreatic cancer OR pancreatic ductal adenocarcinoma) AND (liquid biopsy OR early detection OR biomarker) AND (immune OR immunological reprogramming) [20]. This query was derived through the multiple steps of pilot searches and consensus meetings by author J-c Lee, KR Jung, and EJ Sim. For the possible loss of optimal articles, manual search reviewing citation lists for candidate articles was also performed. Through a cautious literature search and review, we screened, identified, and selected candidate markers that were highly correlated with immunological pathways during the development of PDAC from PanINs. For the initially screened biomarkers which shown more than one time in the literatures, the original articles were reviewed, and the author J-c Lee and KR Jung independently screened the biomarkers based on biological and medical evidence. In cases where their opinions differed, a third author, EJ Sim, participated in a consensus meeting to finalize the selection of biomarkers. The marker-selection scheme is illustrated in Fig. 1.

### Comparison of groups in each stage

In the Fig. 1, the flowchart consists of two steps after literature search phase. In the initial screening step reducing the 55 markers 19 markers, we adopted a ‘negative selection’ approach, and aimed to exclude markers that failed even at this simpler task, comparing non-disease controls with all PC cases (RPC + APC).

In contrast, the second selection step of reducing markers from 19 to 15 involved a ‘positive selection’ strategy, discriminating non-disease control and RPC patients. At this stage, we focused on identifying biomarker panels with actual functional relevance, which required a more conservative and strict evaluation of discriminatory ability.

### Feature and hyperparameter selection

Feature selection and hyperparameter tuning were implemented to minimize the number of biomarkers and generate an optimal machine learning (M/L) model with the best diagnostic performance using ΔCt values as feature variables. The data of all eligible patients were split into training (80%) and test (20%) sets with 4:1 split ratio and controlled randomization, considering the demographic distribution of clinical cancer staging and CA19-9 values to prevent skewed data (Supplementary Figure S1). The test set was not used during the model generation process and only used for the model performance evaluation. When fivefold cross-validation was performed, the training set was further split into sub-training (64%) and validation (16%) sets with 4:1 ratio and controlled randomization for five iterations. Fivefold cross-validation was used to avoid overfitting specific data subsets during the training phase and area under the curve (AUC) was averaged over five validation iterations and used as selection standard. For feature selection process, all possible combinations of markers were analyzed using the logistic regression (LR), random forest (RF), light gradient boost machine (LGBM), extreme gradient boost (XGB), and support vector machine (SVM) with default hyperparameter values unique to each classifier. Twenty models per algorithm with the highest average AUC values were selected for further analysis. 100 models were subjected to hyperparameter fine-tuning while being cross-paired with the remaining algorithms. Consequently, 500 models were subjected to hyperparameter tuning and hyperparameter values with the highest average AUC value of five validation iterations were paired with 500 models. Using each trained model, the predicted probability for each sample was calculated, and for classification, the cut-off that maximized the Youden Index was chosen as the model’s classification threshold.

### Model Building and performance evaluation

Using ΔCt values as feature variables, candidate biomarkers were optimized to produce the best classification performance for distinguishing non-disease controls from the PDAC group. First, feature selection was performed by analyzing all possible biomarker combinations. Using only the training and validation datasets, five-fold cross-validation was conducted using five distinct algorithms suitable for classification: LR, XGB, LGBM, RF, and SVM. Second, the candidate models were subjected to fine-tuning of the hyperparameters, with all possible combinations unique to each algorithm.

For performance evaluation, the training set was used to train the parameter for 500 models and the models were not retrained during the test set evaluation. When CA19-9 was included as part of the model feature, optimal hyperparameter values from the previous process were maintained and the model parameter was trained with the training set prior to performance evaluation using the test set.

Various metrics were employed to evaluate the model performance, including sensitivity, specificity, Youden Index, and AUC, based on the values of True Positive (TP), True Negative (TN), False Positive (FP), False Negative (FN), and receiver operating characteristic (ROC) curve. Sensitivity, specificity, and Youden Index were calculated as follows:

$$Sensitivity\, = \,TP\,/\,\left( {TP\, + \,FN} \right)$$

$$Specificity = \,TN\,/\,\left( {TN\, + \,FP} \right)$$

$$Youden{\text{ }}index\, = \,Sensitivity\, + \,Specificity\, - 1$$

Given the low prevalence of pancreatic cancer, high sensitivity is prioritized over specificity to reduce the risk of missed diagnoses, despite the potential for false positives. Due to sampling bias and the low prevalence of the disease, the specificity of CA19-9 in our dataset may not be clinically relevant. Therefore, performance metrics for HELP-15 were based on AUC, and the Youden Index was used to determine optimal cut-off points for balancing sensitivity and specificity.

Python programming language (version 3.11.4) was used in the feature selection and modeling steps. The Pandas and NumPy libraries were used for data processing, and a sklearn library was used for machine-learning modeling and evaluation.

### Overfitting prevention techniques

To prevent overfitting, we applied the following methods: (1) cross-validation, we performed five-fold cross-validation to enhance generalization performance and prevent the model from overfitting specific data patterns; (2) hyperparameter optimization (grid search), we used GridSearchCV to find the optimal hyperparameters, selecting a model with appropriate complexity to reduce overfitting; (3) normalization and regularization, we applied regularization to each model to reduce overfitting; and (4) optimal cutoff selection, instead of using a simple 0.5 threshold, we maximized the Youden Index to select the optimal cut-off value, balancing sensitivity and specificity.

### Robustness evaluation

The SVM classifier in the HELP-15 model was trained with specific norm regularization to maintain a stable decision boundary, ensuring robustness. We added random noise (0–0.1) to the test data and measured its impact on predictions and hinge loss: Original Hinge Loss = 0.2969; Perturbed Hinge Loss = 0.3011; and Robustness Score (Hinge Loss Difference) = 0.0043. The minimal hinge loss difference (0.0043) confirms that the HELP-15 model remains robust against noise, ensuring stable predictive performance.

### Statistical analysis

The mRNA expression levels (ΔCt) of potential candidate biomarkers were subjected to statistical analyses. Nonparametric tests (Mann–Whitney U and Kolmogorov–Smirnov tests) were performed to select primary biomarker candidates using GraphPad Prism (version 9.5.1; San Diego, CA, USA). Statistical significance was set at *p* < 0.05.

### Data availability

The data generated in this study are available upon reasonable request from the corresponding author.

## Results

### Patient characteristics

In total, 1,963 individuals were identified in this study. After the first exclusion, 1,504 subjects were eligible for the study and divided into two groups: the PDAC group (*n* = 417; 28%) and non-disease control group (*n* = 1, 087; 72%). The PDAC group was further divided into two groups according to clinical staging: RPC (*n* = 76; 5%) and APC (*n* = 341; 22%). The APC group included patients with BRPC/LAPC (*n* = 143; 9%) and MPC (*n* = 198; 13%). The median evaluated CA19-9 levels were significantly different between patients with PDAC and non-disease controls (PDAC, 350.0 U/mL; control, 6.9 U/mL). The non-disease control group consisted of healthy individuals LAPC (*n* = 1020; 93%) and high-risk patients ( *n* = 67; 6%). The demographic data are summarized in Table 1.

**Table 1:** Patient characteristics

	PDAC				Control	Total
		RPC	BRPC /LAPC	MPC		
Number of patients	**417** **(28%)**	76 (5%)	143 (9%)	198 (13%)	1087* (72%)	**1504 †** **(100%)**
Age	**66** **(37**–**88)**	65 (47–88)	66 (38–86)	66 (37–86)	45 (20–76)	**49** **(20**–**88)**
Sex						
male	**219** **(53%)**	48 (63%)	66 (46%)	105 (53%)	689 (63%)	**908** **(60%)**
female	**198** **(48%)**	28 (37%)	77 (54%)	93 (47%)	398 (37%)	**596** **(40%)**
Baseline tumor marker						
CEA	**3.3** **(1**–**1790)**	2.6 (1–142.9)	2.5 (1–118)	5.2 (1–1790)	1.5 (0.3–16)	**1.7** **(0.3**–**1790)**
CA19-9	**350** **(1.9**–**20001)**	109.5 (1.9–7100)	207 (1.9–20001)	1435 (1.9–20001)	6.9 (0.6–84.1)	**8.9** **(0.6**–**20001)**

Data are presented as median (min–max) or *n* (%). PDAC, pancreatic ductal adenocarcinoma; RPC, resectable pancreatic cancer; BRPC, borderline resectable pancreatic cancer; LAPC, locally advanced pancreatic cancer; MPC, metastatic pancreatic cancer; CA19 − 9, carbohydrate antigen 19 − 9; CEA, carcinoembryonic antigen. *Non-disease control: healthy individuals (1020, 93%), long-term diabetes (53, 5%) and chronic pancreatitis (14, 1%). †All 1054 subjects are Korean ethnics

### Candidate marker screening

To search for immunologically relevant markers, a literature search emulating PRISMA flowchart was conducted to identify 1,755 articles and screen 55 biomarkers (Supplementary Table S1). Candidate markers were screened through a series of nonparametric tests to assess the significance of their expression between the PDAC and control groups. Markers with low expression levels (Ct_*GAPDH*_ > 35) during RT-qPCR were excluded from the list of candidates. Consequently, 19 markers remained after screening (Supplementary Figure S2). For the results for the remaining biomarkers from the 55 that were not among the included in the final 19 were shown in Supplementary Figure S3.

### Feature and panel selection

**Fig. 2:**
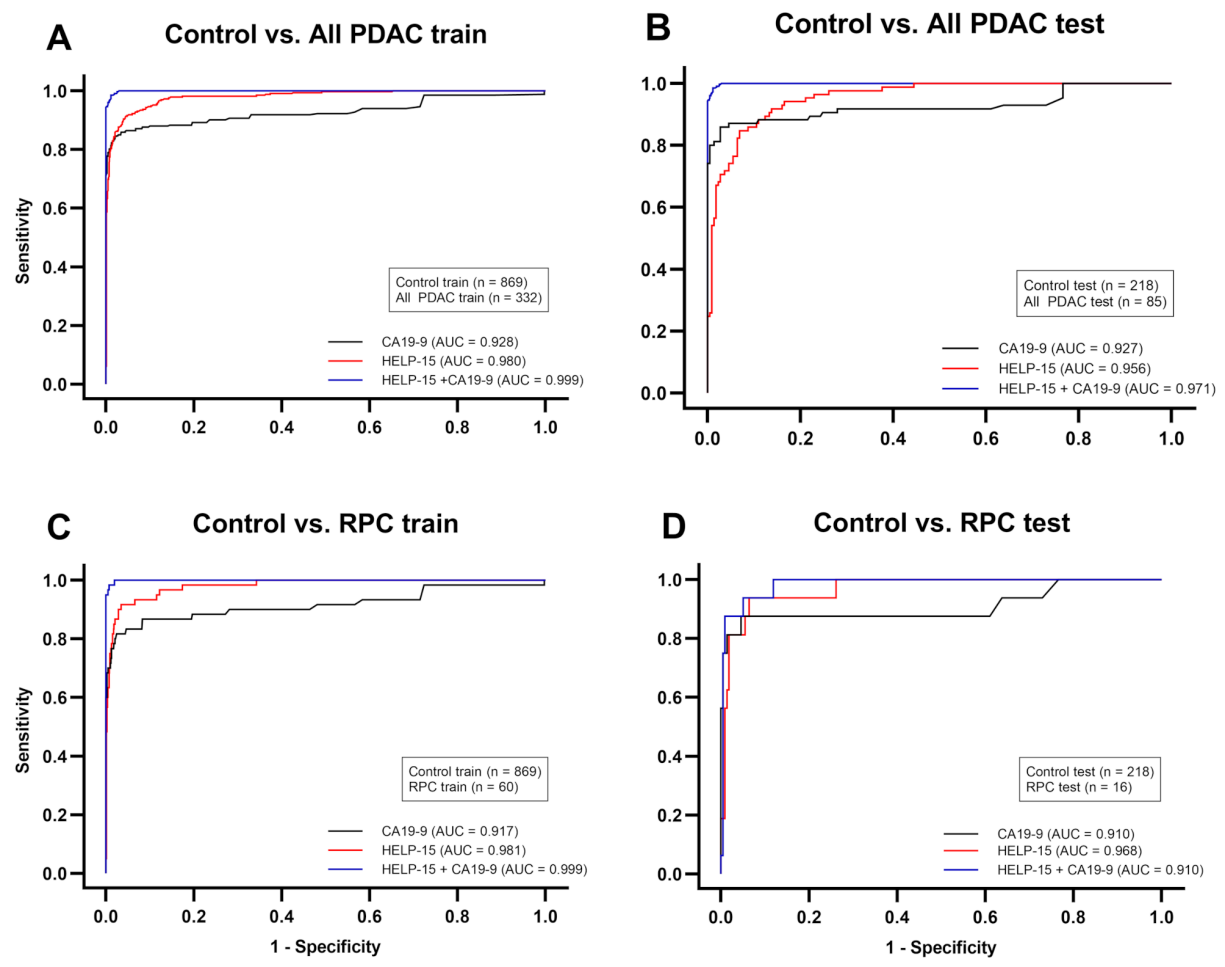
ROC curve for training and test sets of CA19-9 alone,HELP-15, and HELP-15 with CA19-9. **(a)** Control vs. PDAC training set. **(b)** Control vs. PDAC test set. **(c)** Control vs. RPC training set. **(d)** Control vs. RPC test set. CA19-9, carbohydrate antigen 19 − 9; PDAC, pancreatic ductal adenocarcinoma; ROC, receiver operating characteristic; RPC, resectable pancreatic cancer

**Fig. 3:**
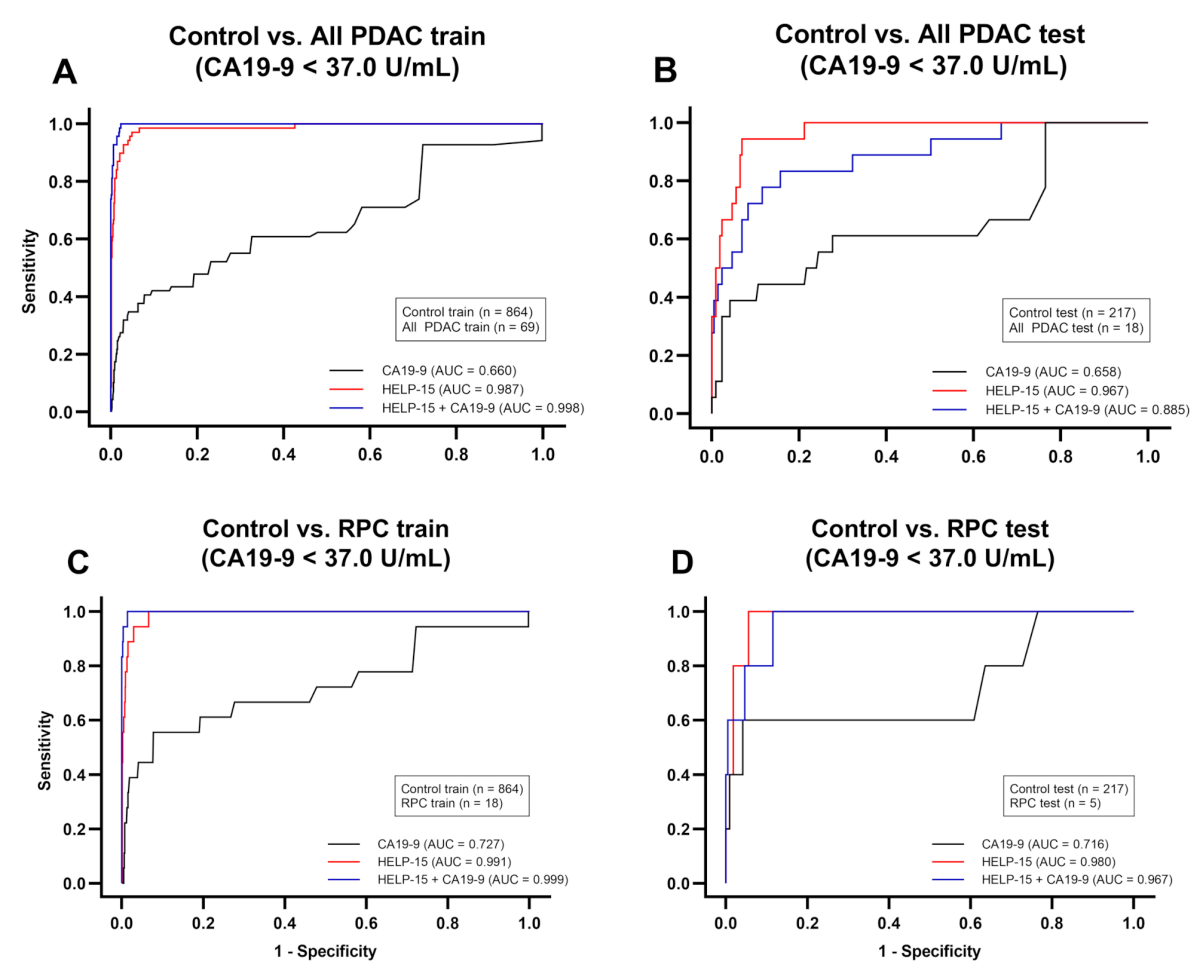
Comparison of diagnostic performance among HELP-15, CA19-9, and their combination for the patients with normal CA19-9 level. Receiver operating characteristic curve for the training and test sets of CA19-9 alone, HELP-15, and HELP-15 with CA19-9. **(a)** Control vs. PDAC training set. **(b)** Control vs. PDAC test set. **(c)** Control vs. RPC training set. **(d)** Control vs. RPC test set. CA19-9, carbohydrate antigen 19 − 9; PDAC, pancreatic ductal adenocarcinoma; RPC, resectable pancreatic cancer

We identified 112 M/L models paired with LR, SVM, XGB, and LGBM0, but except for RF, which surpassed the performance of CA19-9 alone in all four comparisons through performance evaluation. Among them, the SVM algorithm with 15 markers (referred to as HELP-15) was selected as the optimal model for classification as it exhibited the highest AUC value of 0.967 in control vs. PDAC (< 37.0 U/mL; Figs. 2 and 3). In Fig. 2, all the subjects regardless of CA19-9 level were included, whereas only the subjects whose CA19-9 levels were less than 37.0 U/mL were included in Fig. 3. This finding is particularly significant considering the asymptomatic nature of PDAC and false negative results of CA19-9. HELP-15 also achieved an AUC value exceeding 0.950 in all other comparisons. The optimal model included *CCL5*,* CCR5*,* CLEC7A*,* CXCL8*,* CXCR2*,* CXCR4*,* FOXP3*,* IFNA1*,* FNL1*,* PTGES*,* PTGES2*,* PTGS2*,* SLC27A2*,* TNF*, and *VEGFA* as features (Supplementary Table S2).

### Diagnostic performance discriminating PDAC and non-disease control

The AUC of HELP-15 was 0.956 for all PDAC patients, which showed better trend than that of CA19-9 alone (AUC = 0.927) (Table 2). The sensitivity was 82.4%, which was higher than that of CA19-9 alone (76.5%, *P* < 0.001), whereas the specificity was 93.6%, which was lower than that of CA19-9 alone (99.5%, *P* = 0.5).

**Table 2:** Diagnostic performance of the marker panel

		Training set				Test set			
	Marker panel	Sensitivity (95% CI)	Specificity (95% CI)	AUC (95% CI)	*p*-value (Area = CA19-9 only AUC)	Sensitivity (95% CI)	Specificity (95% CI)	AUC (95% CI)	*p*-value (Area = CA19-9 only AUC)
**PDAC**	**CA19-9 only**	78.9 (74.1–83.2)	99.4 (98.7–99.8)	0.928 (0.905–0.950)	N/A	76.5 (66.0–85.0)	99.5 (97.5–100.0)	0.927 (0.880–0.965)	N/A
	**15 markers**	91.3 (87.7–94.1)	95.6 (93.9–96.8)	0.980 (0.973–0.988)	*< 0.001*	82.4 (72.6–89.8)	93.6 (88.9–96.1)	0.956 (0.933–0.979)	***0.249***
	**15 markers + CA19-9**	98.2 (96.1–99.3)	98.8 (97.9–99.4)	0.999 (0.998-1.000)	*< 0.001*	92.9 (85.3–97.4)	92.2 (87.8–95.4)	0.971 (0.949–0.994)	*0.014*
**RPC**	**CA19-9 only**	70.0 (56.8–81.2)	99.4 (98.7–99.8)	0.917 (0.855–0.967)	N/A	62.5 (35.4–84.8)	99.5 (97.4–100.0)	0.910 (0.773–0.998)	N/A
	**15 markers**	91.7 (81.6–97.2)	95.6 (93.9–96.8)	0.981 (0.967–0.995)	*0.030*	93.8 (69.8–99.8)	93.6 (88.9–96.1)	0.968 (0.935–1.000)	*0.334*
	**15 markers + CA19-9**	98.3 (91.1–100.0)	98.8 (97.9–99.4)	0.999 (0.998-1.000)	*0.003*	93.8 (69.8–99.8)	92.2 (87.8–95.4)	0.985 (0.968–1.000)	*0.150*
**PDAC** **(CA19-9 < 37.0 U/ml)**	**CA19-9 only**	N/A	N/A	0.660 (0.585–0.741)	N/A	N/A	N/A	0.658 (0.509–0.802)	N/A
	**15 markers**	95.7 (87.8–99.1)	95.6 (93.9–96.7)	0.987 (0.974–0.999)	*< 0.001*	88.9 (65.3–98.6)	93.5 (88.9–96.0)	0.967 (0.939–0.994)	*< 0.001*
	**15 markers + CA19-9**	91.3 (82.0-96.7)	99.4 (98.7–99.8)	0.998 (0.996–1.000)	*< 0.001*	66.7 (41.0–86.7)	92.6 (88.3–95.7)	0.885 (0.795–0.974)	*0.001*
**RPC** **(CA19-9 < 37.0 U/ml)**	**CA19-9 only**	N/A	N/A	0.727 (0.562–0.873)	N/A	N/A	N/A	0.716 (0.331–0.995)	N/A
	**15 markers**	94.4 (72.7–99.9)	95.6 (93.9–96.8)	0.991 (0.983–0.999)	*< 0.001*	100.0 (47.8–100.0)	93.5 (88.9–96.1)	0.980 (0.957–1.000)	*0.099*
	**15 markers + CA19-9**	94.4 (72.7–99.9)	99.4 (98.7–99.8)	0.999 (0.997–1.000)	*< 0.001*	80.0 (28.4–99.5)	92.6 (88.3–95.7)	0.967 (0.921–1.000)	*0.090*

AUC, area under curve; PDAC, pancreatic ductal adenocarcinoma; RPC, resectable pancreatic cancer; CA 19 − 9, carbohydrate antigen 19 − 9. Sensitivity and specificity 95% confidence intervals (CIs) were calculated using the Clopper-Pearson CI. AUC 95% confidence intervals (CIs) and *p*-values were calculated using the DeLong Test

More detailed analysis in dividing APC group (among BRPC/LAPC, MPC) is shown in Supplementary table S3. Additional diagnostic performance analyses were conducted for BRPC/LAPC and MPC. For the CA19-9 marker alone, specificity was found to be the highest in both BRPC/LAPC and MPC cohorts. However, the HELP-15 marker panel outperformed other models in terms of sensitivity and AUC. Furthermore, the HELP-15 marker panel demonstrated consistently superior performance in both BRPC/LAPC and MPC, regardless of CA19-9 levels.

In an analysis of data with CA19-9 levels < 37.0, the 16-marker model incorporating CA19-9 exhibited a sensitivity of 0 for the BRPC/LAPC label. This issue, which is presumed to be caused by data imbalance, likely resulted from the limited number of data points (*n* = 5) with CA19-9 levels < 37.0 labeled as BRPC/LAPC, thereby negatively impacting the evaluation of model performance.

For patients with RPC, the biomarker panel showed an AUC of 0.968, which was higher than that of CA19-9 (AUC = 0.910). The sensitivity was 93.8%, which was significantly higher than that of CA19-9 alone (62.5%, *P* < 0.001), although the specificity (93.6%) was lower than that of CA19-9 alone (99.5%, *P* = 0.040). HELP-15 showed great improvements over CA19-9 in distinguishing PDAC from the control group, especially in patients with RPC and normal level of CA19-9.

### Additional analysis of patients with normal CA19-9 levels

The general clinical cut-off value for the CA19-9 test was 37.0 U/mL. It is recommended that individuals with CA19-9 levels ≥ 37.0 U/mL are recommended to proceed with imaging techniques and continuous observation. In this study, 21% of patients with PDAC had normal CA19-9 levels (< 37.0 U/mL). Considering the genetic absence and delayed expression of CA19-9 in the population, patients with PDAC and normal CA19-9 levels are important targets for improving the overall PDAC diagnosis. The sensitivity and specificity of CA19-9 alone for patients with CA19-9 levels < 37.0 U/mL were not calculated because all data fell below the clinical standard. Only the AUC values were compared with those of the final model.

When comparing patients between the control and PDAC groups with normal CA19-9 levels, the sensitivity, specificity, and AUC of HELP-15 were 88.9% (*P* < 0.001), 93.5% (*P* < 0.001), and 0.967 (*P* < 0.001), respectively (Fig. 3; Table 2). The AUC of CA19-9 alone was 0.658, which was significantly lower than that of the final model. For patients with RPC and normal CA19-9 levels, the sensitivity, specificity, and AUC of the final model were 100.0%, 93.5%, and 0.980, respectively. Using the same test set, CA19-9 alone showed an AUC of 0.716, which was significantly lower than that of the final model.

### Feature importance analysis for HELP-15 panel

To investigate the weight of importance of each feature in HELP-15 panel, we added the complete feature importance analysis results in Supplementary Fig S4, Fig S5, and Supplementary Table S4. Figure S4 visually represents the relative importance of each marker, while Figure S5 and Table S4 demonstrate that maintaining all markers is essential for optimizing performance of the HELP-15 panels.

## Discussion

We developed an immune system-derived mRNA biomarker panel accompanied by an M/L algorithm that displayed effective diagnostic performance against PDAC. The panel included *CCL5*,* CCR5*,* CLEC7A*,* CXCL8*,* CXCR2*,* CXCR4*,* FOXP3*,* IFNA1*,* IFNL1*,* PTGES*,* PTGES2*,* PTGS2*,* SLC27A2*,* TNF*, and *VEGFA*, which were selected from 55 candidate biomarkers using a series of nonparametric tests, M/L-based feature selections, and performance evaluations. Based on these results, we constructed an optimal biomarker model that showed improvements in distinguishing between the RPC and PDAC groups with normal CA19-9 levels.

In the last two decades, several studies have focused on combining multiple proteins, miRNAs, and cfDNAs to achieve meaningful PDAC diagnostic performance [10–13, 21–23]. Klein et al. demonstrated a reasonable performance for various cancer types. However, the sensitivity for PDAC was measured at 83.7%, with a lower stage I sensitivity of 61.9% [24]. Lee et al. reported a sensitivity of 92.5% for detecting RPC; however, the sensitivity was 64.3% in the group with a normal range of CA19-9 when using triple protein marker panels, including LRG1, TTR, and CA19-9 [25]. Recently, Nakamura et al. showed excellent diagnostic performance with their transcriptomic signature, with an AUC of 0.930 and sensitivity in the early stages of PDAC (stages I and II) [26]. However, exosome-based and cell-free miRNAs require complicated analytical procedures that use unstable exosome-based miRNAs, making signatures less accessible to the general population [27]. Therefore, clinically feasible biomarkers are needed for PDAC detection.

In this context, our biomarker panel demonstrated several notable strengths compared to those in previous studies. First, the final model consistently outperformed CA19-9 in diagnosing RPC (Fig. 2c and d), even in patients with normal CA19-9 levels (Fig. 3). Second, the use of RT-qPCR, a widely available and cost-effective analytical method, enhances the accessibility and affordability of the HELP-15 panel for the general population. Third, we utilized machine-learning models to optimize the diagnostic performance of the final model by leveraging extensive clinical data. By partitioning the dataset into training, validation, and test sets, we minimized the risk of overfitting and ensured robust performance. The efficacy of the biomarker panel was particularly promising for patients with RPC and normal CA19-9 levels. Although the CA19-9 test is limited by its delayed expression in early-stage PDAC and susceptibility to false-negative results, the HELP-15 panel, when used in conjunction with the CA19-9 test, shows significant potential as a screening tool for PDAC. Although biologically confirmed interactive model between genetic and immunological mapping in PDAC tumorigenesis, we suggest an immune system reprogramming model in early PDAC (shown in Supplementary Figure S6, table S1).

In the process of selecting HELP-15, we prioritized sensitivity over specificity for the following reasons. In general health check-ups, HELP-15 would replace CA19-9, where specificity is key to distinguish pancreatic cancer from other cancers. However, in the other scenarios where pancreatic cancer is already clinically suspected (such as periodic surveillance in patients with high risk group, or monitoring minimal residual disease and early recurrence after PDAC resection), specificity is already ensured by clinical suspicion—the primary focus should be on sensitivity for early detection. Since our HELP-15 model was developed as a potential replacement or complement to CA19-9 in the second scenario, we have prioritized sensitivity over specificity.

Our study had some limitations. First, there was a disproportionate age distribution between individuals in the PDAC and control groups. To ensure that HELP-15 was not biased by age, we performed a correlation test between age and model probability, which indicated that the correlation was not significant (Supplementary Figure S7). Also we added more correlation test for male and female (Supplementary Figure S8). Second, some molecular biological mechanisms have not been fully elucidated. However, we implemented a structured representation method that included a literature review emulating PRISMA flowchart and multifaceted comparison of the experimental data. Third, in the phase of literature review, there is a lack of quantitative evaluation for selecting biomarkers. However, it is important to note that systematic reviews or meta-analyses (or the reviews emulating SR-MA) inherently involve a certain level of subjectivity. To overcome this limitation, the original articles were reviewed by two authors independently and third author held consensus meetings for discrepancy between two authors. Fourth, the prevalence of PDAC in the cohort may not accurately reflect the real-world PDAC prevalence. This can be applied to both overall PDAC prevalence and proportion of each stage. Therefore, despite of the valuable insights of this study, the results should be interpreted with caution, considering the potential differences in prevalence and the generalizability of the model to a wider population. Finally, the study was conducted at a single center. While multicenter research has its advantages, a homogeneous population might be more effective for identifying novel biomarkers. However, samples collected from a single site especially in a homogenous ethnic group may introduce site bias, which cannot be excluded and could be a significant limitation. Therefore, further large-scale and multicenter clinical validation is needed to overcome these potential biases and strengthen the generalizability of the findings.

In conclusion, compared with CA19-9, the immune system-derived mRNA biomarker panel, HELP-15, showed better trend than CA19-9 in discriminating PDAC. Especially in RPC patients with normal CA19-9 level, HELP-15 significantly improved the diagnostic performance, which can be considered as a promising marker for the early detection of RPC.

## Electronic supplementary material

Below is the link to the electronic supplementary material.

Supplementary Material 1

Supplementary Material 2

Supplementary Material 3

Supplementary Material 4

Supplementary Material 5

Supplementary Material 6

Supplementary Material 7

Supplementary Material 8

Supplementary Material 9

Supplementary Material 10

## Data Availability

The data that support the findings of this study are not openly available due to reasons of sensitivity and are available from the corresponding author upon reasonable request. Data are located in controlled access data storage at the Human Bioresource Center of Seoul National University Bundang Hospital.

## Abbreviations

APC: Advanced pancreatic cancer
AUC: Area under the curve
BRPC: Borderline resectable pancreatic cancer
CA19-9: Carbohydrate antigen 19 − 9
FN: False negative
FP: False positive
LAPC: Locally advanced pancreatic cancer
LGBM: Light gradient boost machine
LR: Logistic regression
M/L: Machine learning
MPC: Metastatic pancreatic cancer
PDAC: Pancreatic ductal adenocarcinoma
RF: Random forest
RPC: Resectable pancreatic cancer
SVM: Support vector machine
TME: Tumor microenvironment
TN: True negative
TP: True positive
XGB: extreme gradient boost
