# Supplementary material for: Novel mRNA biomarker-based liquid biopsy for the detection of resectable pancreatic cancer
Source: BMC Cancer. 2025 Apr 23;25:762. doi: 10.1186/s12885-025-14124-w (PMC12016232; doi:10.1186/s12885-025-14124-w)
Supplement: Supplementary file 1 — Supplementary Material 1 [file 12885_2025_14124_MOESM1_ESM.docx]

**Supplementary Data**

**Novel mRNA Biomarker-based Liquid Biopsy for the Detection of Resectable Pancreatic Cancer**

**Jong-chan Lee^1,2,†^, Sung Won Kang^3,4,†^, Eun-Jin Sim^3^, Jin-Sik Bae^3^, Seong-mo Koo^3^, Mun-sub Byoun^3^, Serin Kwon^5^, Seoi Hong^6^, Yunji Kim^6^, Yuna Youn^1^, Kwangrok Jung^1^, Jaihwan Kim^1,2^, Hyoung Hwa Jeong^3^, Jihie Kim^6^*, Jin-Hyeok Hwang^1,2^***

^1^Department of Internal Medicine, Seoul National University Bundang Hospital, Seongnam, Korea

^2^Department of Internal Medicine, Seoul National University College of Medicine, Seoul, Korea

^3^Research Center, HuVet bio Inc., Seoul, Korea

^4^Department of Translational Medicine, Seoul National University College of Medicine, Seoul, Korea

^5^Department of Life Science, Dongguk University, Seoul, Korea

^6^Department of Computer and Artificial Intelligence, Dongguk University, Seoul, Korea

^†^These authors contributed equally to this work as co-first authors

^*^These authors contributed equally to this work as co-corresponding authors

**Supplementary Figure Legends**

**Supplementary Figure S1**. Schematic representation of the controlled randomization of patient data. Numbers below the total sample size represents the split numbers of the training, validation, and test sets with the ratio of 6.4:1.6:2.0, respectively.

**Supplementary Figure S2.** Differences in mRNA expression levels of 19 candidate markers between the control and PDAC groups. The mRNA expression level was calculated as the ΔCt (Ct_target gene_ – Ct*_GAPDH_*). Increased ΔCt indicates a decreased expression level, and vice versa. The p-values were calculated using Mann–Whitney (*p <0.05, **p <0.01, ***p <0.001, and ****p <0.0001) and Kolmogorov–Smirnov (†p <0.05, ††p <0.01, †††p <0.001, and ††††p <0.0001) tests. **(A)** Upregulated expression levels. **(B)** Downregulated expression levels. **(C)** Distribution changes in expression levels.

**Supplementary Figure S3.** Differences in mRNA expression levels of 23 markers which showed non-significant expression difference between the control and PDAC groups. The mRNA expression level was calculated as the ΔCt (Ct_target gene_ – Ct*_GAPDH_*). Increased ΔCt indicates a decreased expression level, and vice versa. The p-values were calculated using Mann–Whitney Kolmogorov–Smirnov tests. 20 each of Control and PDAC samples was used.

**Supplementary Figure S4.** The following is a graph that calculates and visualizes the Feature Importance of each marker in the HELP-15 panel using the Permutation Importance method. The Permutation Importance method evaluates the model's performance based on AUC and then recalculates the performance after randomly shuffling each feature data. This approach allows for a quantitative understanding of how much each marker contributes to the model's performance. The graph visualizes the impact of the 15 markers in the HELP-15 panel on the model's performance at a glance.

**Supplementary Figure S5.** This illustrates how model performance changes depending on the Feature Importance Threshold. The X-axis of the graph represents the Feature Importance Threshold, while the Y-axis indicates the performance metric. Based on the specified threshold, markers with feature importance below the threshold were excluded, and the model's performance was evaluated using the remaining markers. The results demonstrate that as the number of markers in the panel decreases, the model's performance declines. This finding highlights that the current marker panel, HELP-15, is the optimal marker panel, exhibiting the best performance across all aspects, including AUC, Sensitivity, and Specificity.

**Supplementary Figure S6**. Suggested mechanism for immune system reprogramming in the early phase of pancreatic cancer and interactions of candidate markers. In the early phase of PDAC development, the epithelial-to-mesenchymal transition (EMT) occurs in precancerous pancreatic cells. This mechanism allows circulating tumor cells (CTCs) to be detected in blood vessels (1-6). These CTCs could be precursors of metastasis, which can be found as CTC clusters composed of non-malignant cells, such as mesenchymal stem and immune cells (7). During the circulation of CTC clusters in the bloodstream, cancer-associated mesenchymal stem cells (CA-MSCs) promote the proliferation, invasion, and metastasis of PDAC and induce immunosuppressive polarization in immune cells (8). In particular, granulocyte macrophage colony-stimulating factor (GM-CSF) secreted by CA-MSCs induces the transformation of neutrophils into polymorphonuclear myeloid-derived suppressor cells (PMN-MDSCs) through GM-CSF/STAT5/SLC27A2 pathway-mediated arachidonic acid uptake, which ultimately leads to prostaglandin E2 (PGE2)-mediated CD8+ T cell suppression (9-12). In this process of immune system reprogramming, various types of signaling pathways are involved (**Supplementary Figure S1**). However, such immunological changes may have distinct characteristics between early and advanced PDAC. In the present study, we aimed to identify novel immunologic markers for PC diagnosis with a focus on PC-specific early reprogramming of the immune system, which is substantially associated with tumorigenesis in resectable PDAC.

**Supplementary Figure S7**. Scatter plot of the control and PDAC groups with age and model probability. Spearman correlation test showing p-values of 0.1434 and 0.8208 in the control and PDAC groups, respectively.

**Supplementary Figure S8**. Statistical analysis results of HELP-15 biomarkers and model probability (male vs. female) shows no definite bias according to sex.

**Supplementary References**

1. Fan Z, Fan K, Yan C, Huang Q, Gong Y, Cheng H, *et al*. Critical role of KRAS mutations in pancreatic ductal adenocarcinoma. *Transl Cancer Res* **2018 Oct**7:1728–36. doi: [10.21037/tcr.2018.10.19](https://doi.org/10.21037/tcr.2018.10.19).

2. Roy N and Hebrok M. Regulation of cellular identity in cancer. *Dev Cell* **2015 Dec 21**;35:674–84. doi: [10.1016/j.devcel.2015.12.001](https://doi.org/10.1016/j.devcel.2015.12.001).

3. Chuvin N, Vincent DF, Pommier RM, Alcaraz LB, Gout J, Caligaris C, *et al.* Acinar-to-ductal metaplasia induced by transforming growth factor-beta facilitates KRASG12D-driven pancreatic tumorigenesis. *Cell Mol Gastroenterol Hepatol* **2017 May 31**;4:263–82. doi:[10.1016/j.jcmgh.2017.05.005](https://doi.org/10.1016/j.jcmgh.2017.05.005).

4. Hanahan D and Weinberg RA. Hallmarks of Cancer: The Next Generation. *Cell* **2011 Mar 4**;144:646–74. doi: [10.1016/j.cell.2011.02.013](https://doi.org/10.1016/j.cell.2011.02.013).

5. Polyak K, Weinberg RA. Transitions between the epithelial and mesenchymal states: Acquisition of malignant and stem cell traits. *Nat Rev Cancer* **2009 Apr**;9:265–73. doi: [10.1038/nrc2620](https://doi.org/10.1038/nrc2620).

6. Rhim AD, Mirek ET, Aiello NM, Maitra A, Bailey JM, McAllister F, *et al*. EMT) and dissemination precede pancreatic tumor formation. *Cell* **2012 Jan 20**;148:349–61. doi: [10.1016/j.cell.2011.11.025](https://doi.org/10.1016/j.cell.2011.11.025).

7. Hong Y, Fang F, Zhang Q. Circulating tumor cell clusters: what we know and what we expect (Review) (review). *Int J Oncol* **2016 Dec**;49:2206–16. doi: [10.3892/ijo.2016.3747](https://doi.org/10.3892/ijo.2016.3747).

8. Shi Y, Du L, Lin L, Wang Y. Tumor-associated mesenchymal stem/stromal cells: emerging therapeutic targets. *Nat Rev Drug Discov* **2017 Jan**;16:35–52. doi: [10.1038/nrd.2016.193](https://doi.org/10.1038/nrd.2016.193).

9. Sai B, Dai Y, Fan S, Wang F, Wang L, Li Z, *et al.* Cancer-educated stem cells promote the survival of cancer cells at primary and distant metastatic sites via the expansion of bone marrow-derived PMN-MDSCs. *Cell Death Dis* **2019 Dec 9**;10:941. doi:[10.1038/s41419-019-2149-1](https://doi.org/10.1038/s41419-019-2149-1).

10. Mathew E, Brannon AL, Del Vecchio AC, Garcia PE, Penny MK, Kane KT, *et al.* stem cells promote pancreatic tumor growth by inducing alternative polarization of macrophages. *Neoplasia* **2016 Mar**;18:142–51. doi: [10.1016/j.neo.2016.01.005](https://doi.org/10.1016/j.neo.2016.01.005).

11. Waghray M, Yalamanchili M, Dziubinski M, Zeinali M, Erkkinen M, Yang H, *et al.* GM-CSF mediates mesenchymal-epithelial cross-talk in pancreatic cancer. *Cancer Discov* **2016 Aug**;6:886–99. doi: [10.1158/2159-8290.CD-15-0947](https://doi.org/10.1158/2159-8290.cd-15-0947).

12. Veglia F, Tyurin VA, Blasi M, De Leo A, Kossenkov AV, Donthireddy L, *et al*. Fatty acid transport protein 2 reprogrammed neutrophils in cancer cells *Nature* **2019 May**;569:73–8. doi: [10.1038/s41586-019-1118-2](https://doi.org/10.1038/s41586-019-1118-2).
