# Supplementary figures and images for: Novel mRNA biomarker-based liquid biopsy for the detection of resectable pancreatic cancer
Source: BMC Cancer. 2025 Apr 23;25:762. doi: 10.1186/s12885-025-14124-w (PMC12016232; doi:10.1186/s12885-025-14124-w)

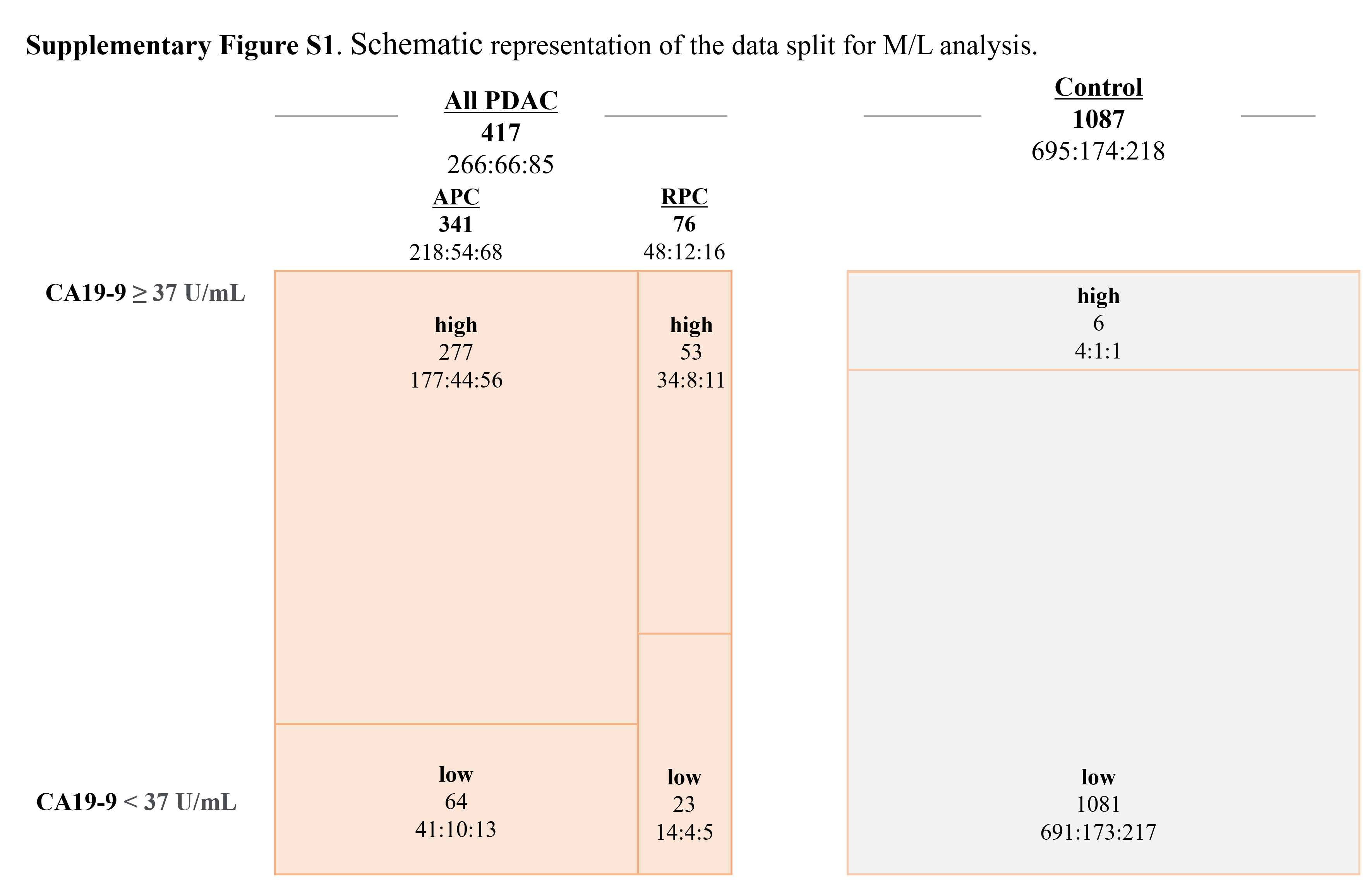

Supplement: Supplementary file 3 — Supplementary Material 3 [file 12885_2025_14124_MOESM3_ESM.tif]

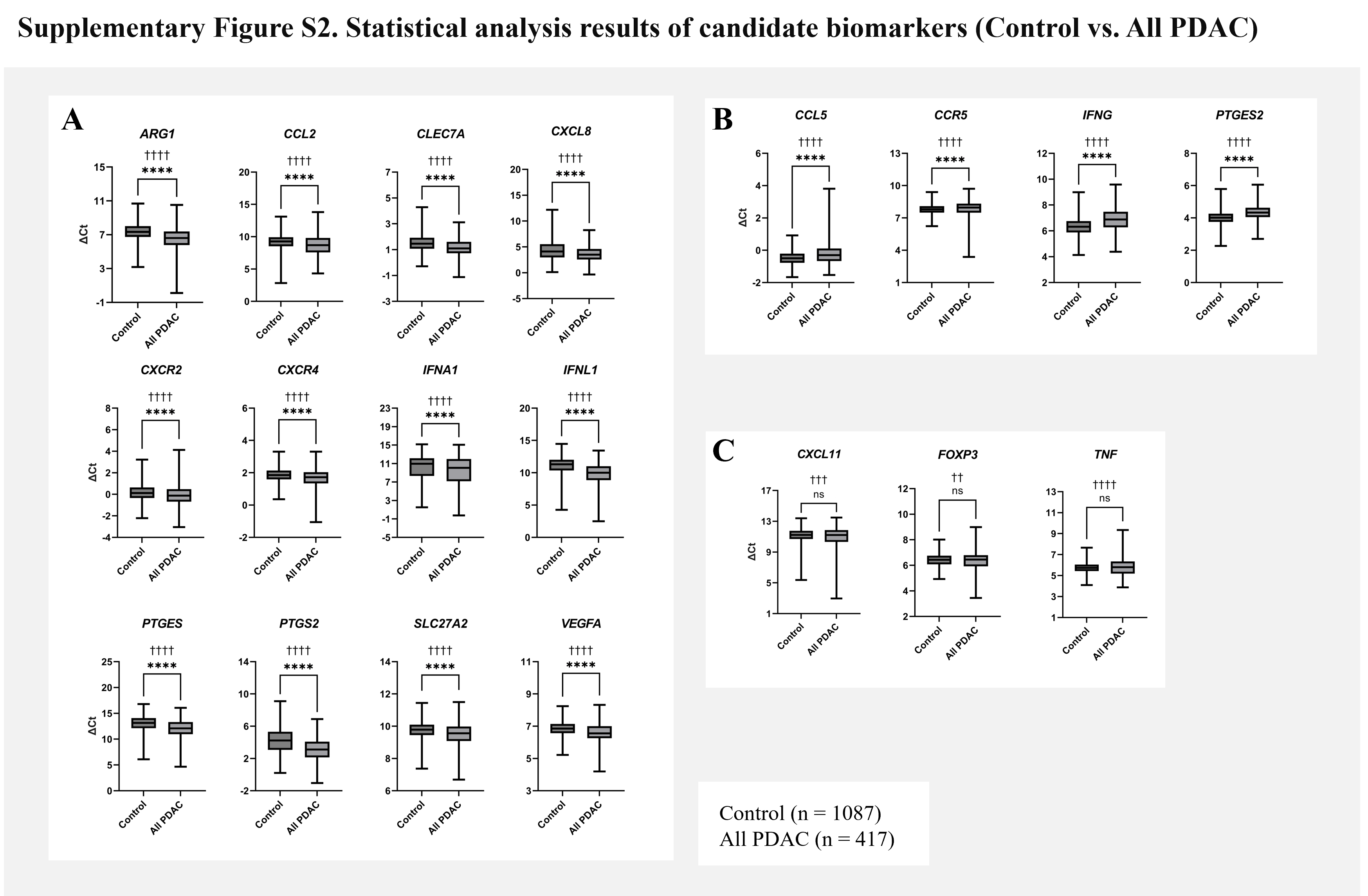

Supplement: Supplementary file 4 — Supplementary Material 4 [file 12885_2025_14124_MOESM4_ESM.tif]

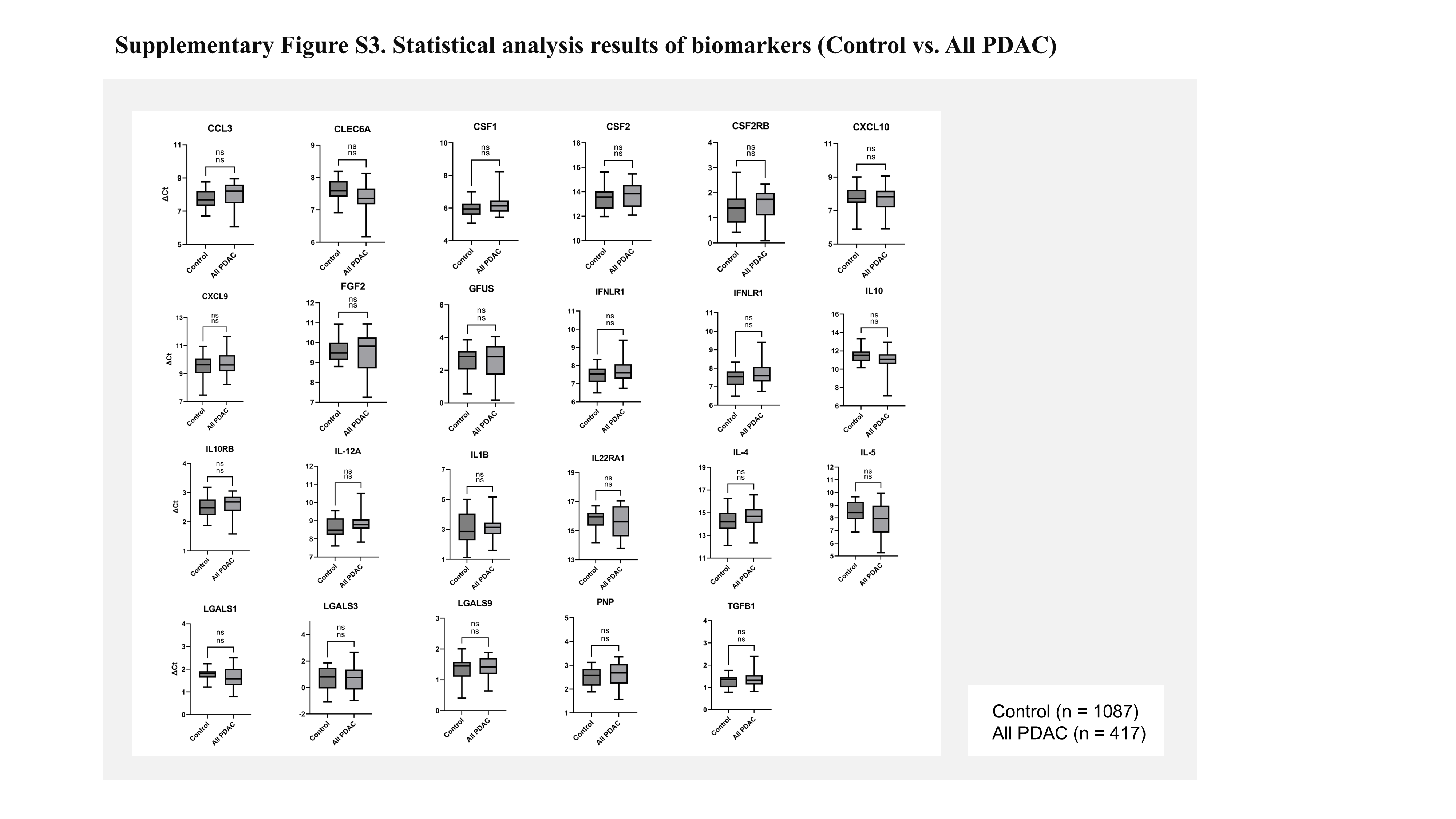

Supplement: Supplementary file 5 — Supplementary Material 5 [file 12885_2025_14124_MOESM5_ESM.tif]

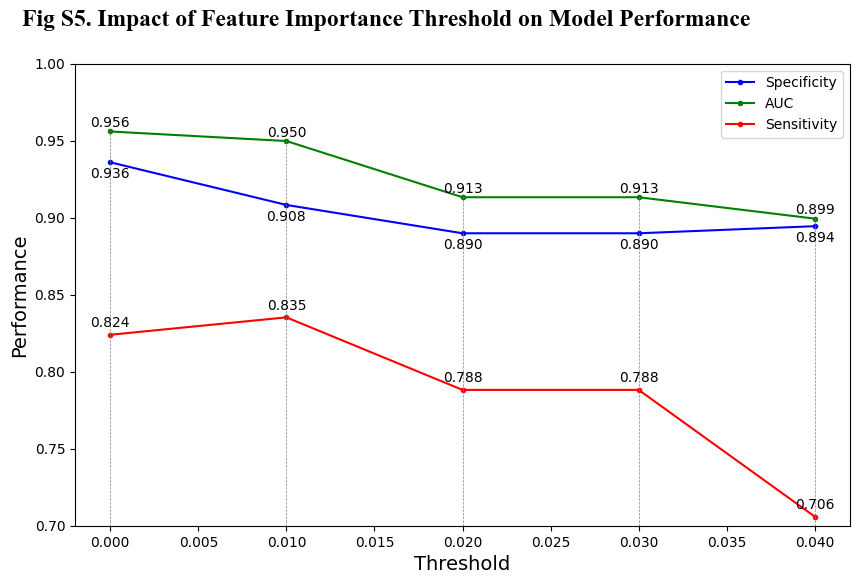

Supplement: Supplementary file 7 — Supplementary Material 7 [file 12885_2025_14124_MOESM7_ESM.tif]

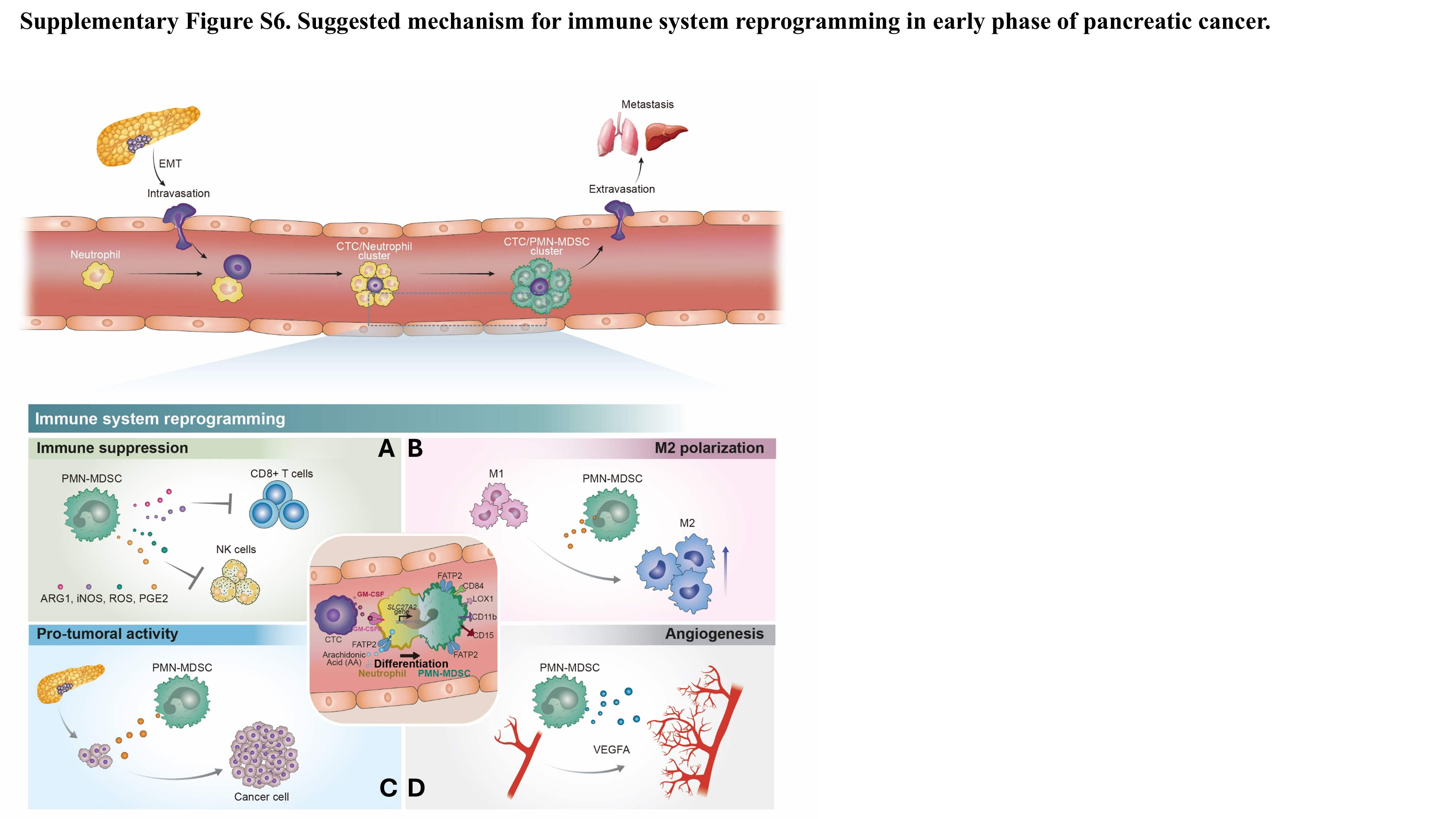

Supplement: Supplementary file 8 — Supplementary Material 8 [file 12885_2025_14124_MOESM8_ESM.tif]

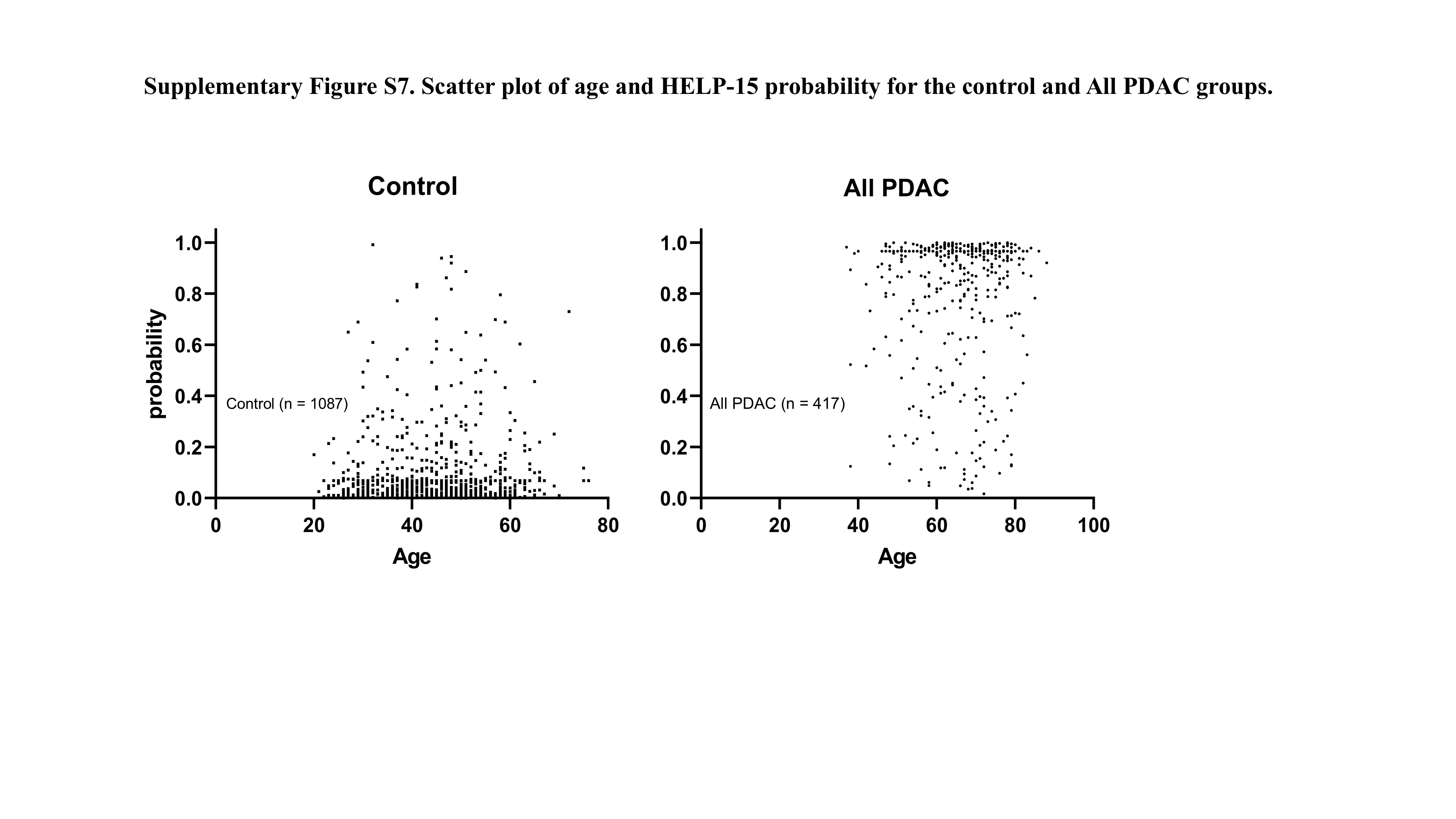

Supplement: Supplementary file 9 — Supplementary Material 9 [file 12885_2025_14124_MOESM9_ESM.tif]

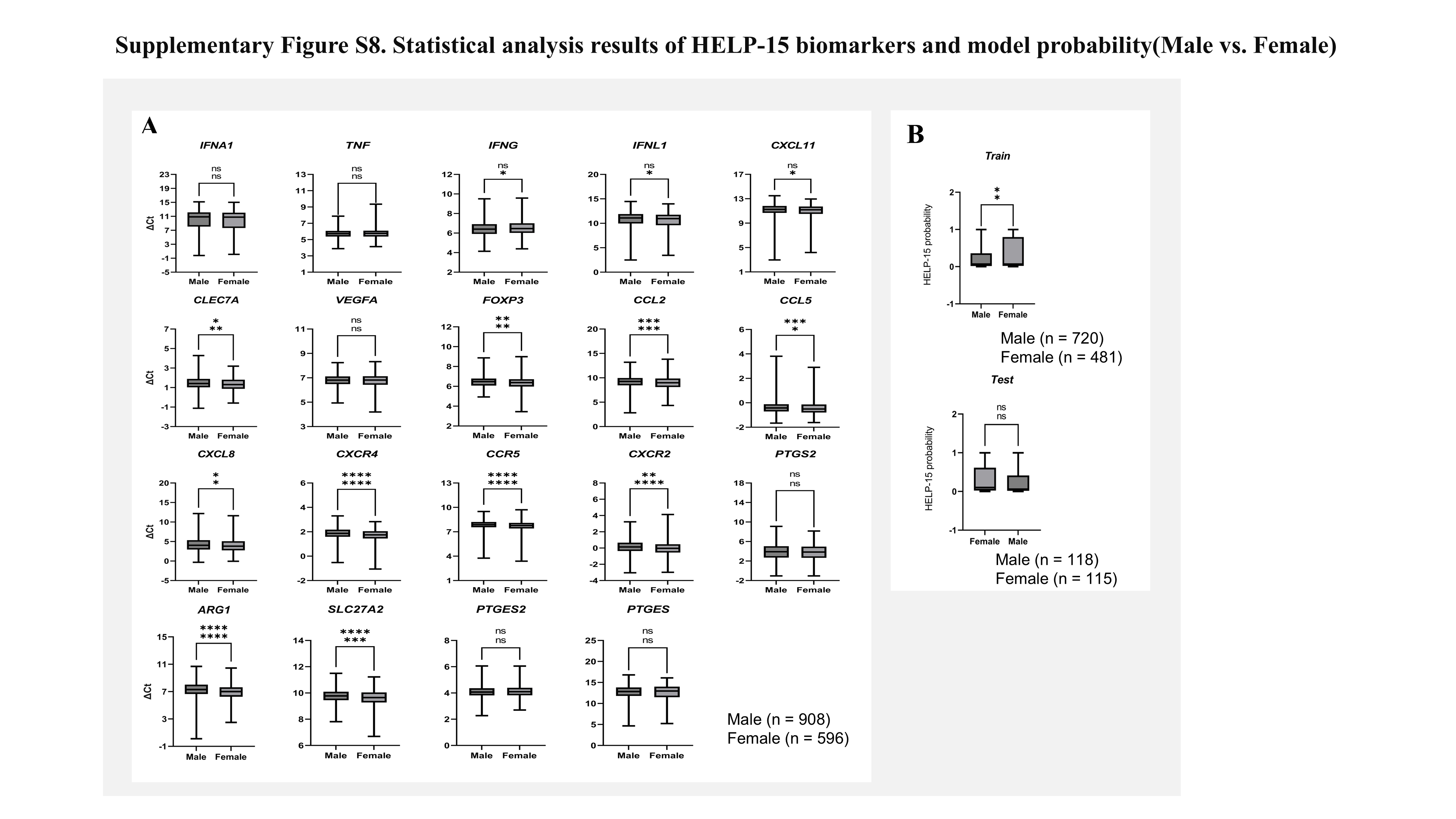

Supplement: Supplementary file 10 — Supplementary Material 10 [file 12885_2025_14124_MOESM10_ESM.tif]
